# Features of the oral microbiome in Japanese elderly people with 20 or more teeth and a non-severe periodontal condition during periodontal maintenance treatment: A cross-sectional study

**DOI:** 10.3389/fcimb.2022.957890

**Published:** 2022-10-06

**Authors:** Naoki Toyama, Daisuke Ekuni, Aya Yokoi, Daiki Fukuhara, Md Monirul Islam, Nanami Sawada, Yukiho Nakashima, Momoko Nakahara, Ichiro Sumita, Manabu Morita

**Affiliations:** ^1^ Department of Preventive Dentistry, Faculty of Medicine, Dentistry and Pharmaceutical Sciences, Okayama University, Okayama, Japan; ^2^ Department of Preventive Dentistry, Okayama University Hospital, Okayama, Japan

**Keywords:** oral microbiome, elderly people, diversity, bacteria, non-severe periodontal condition

## Abstract

**Introduction:**

The aim of the present study was to characterize the profile and diversity of the oral microbiome of a periodontally non-severe group with ≥20 teeth in comparison with a severe periodontitis group of elderly Japanese people.

**Methods:**

A total of 50 patients who had ≥20 teeth and aged ≥60 years were recruited, and 34 participants (13 non-severe participants) were analyzed. After oral rinse (saliva after rinsing) sample collection, the V3–V4 regions of the 16S rRNA gene were sequenced to investigate microbiome composition, alpha diversity (Shannon index, Simpson index, richness, and evenness), and beta diversity using principal coordinate analysis (PCoA) based on weighted and unweighted UniFrac distances. A linear discriminant analysis effect size was calculated to identify bacterial species in the periodontally non-severe group.

**Results:**

The periodontally non-severe group showed lower alpha diversity than that of the severe periodontitis group (p <0.05); however, the beta diversities were not significantly different. A higher relative abundance of four bacterial species (*Prevotella nanceiensis*, *Gemella sanguinis*, *Fusobacterium periodonticum*, and *Haemophilus parainfluenzae*) was observed in the non-severe group than that in the severe periodontitis group.

**Conclusion:**

The oral microbiome in elderly Japanese people with ≥20 teeth and a non-severe periodontal condition was characterized by low alpha diversity and the presence of four bacterial species.

## 1 Introduction

Prevention of tooth loss is important for maintaining a good quality of life in elderly people. The number of remaining teeth affects dietary intake (Yoshihara et al., 2005). Since the number of remaining teeth is associated with successful aging in older Japanese people (Tanji et al., 2020), the 8020 Campaign (keeping 20 teeth or more at 80 years of age) was proposed (8020 Promotion Foundation, 2021). The 8020 Campaign is based on data showing that elderly people with ≥20 teeth can eat food without difficulty (Yoshihara et al., 2005). The proportion of elderly people with ≥20 teeth is increasing.

The next challenge is to increase the number of teeth with good periodontal condition. The prevalence of periodontitis is about 20%–50% in the global population (Nazir, 2017), and severe periodontitis has increased over the past 30 years (Chen et al., 2021). In Japan, the National Survey of Dental Diseases in 2016 showed that the prevalence of pocket depth ≥4 mm was 44.1%–57.9% in elderly people (≥60 years old) (Survey of Dental Diseases, 2017). Periodontitis can lead to the worsening of diabetes mellitus, coronary heart disease, stroke, rheumatoid arthritis, cancer, chronic kidney disease, and respiratory disease and increase the risk of preterm delivery (Nazir, 2017). In addition, the loss of periodontal support structures has a negative impact on masticatory cycle efficiency and molar bite force (Palinkas et al., 2019). Nielsen et al. (2016) showed the negative effects of periodontitis on dietary intake regardless of the number of remaining teeth. Therefore, to maintain the quality of life of the elderly, it is important for the remaining teeth to have good periodontal health.

Understanding the characteristics of the oral microbiome is important for understanding periodontitis. Periodontitis is caused by oral bacteria. The oral microbiome is a bacterial community that consists of over 600 bacterial species (Dewhirst et al., 2010). Of all the bacteria in humans, 26% are present in the mouth (Peterson et al., 2009). The characteristics of the oral microbiome, including microbial profile and diversity, critically affect the maintenance of good health or progression toward disease (Mark Welch et al., 2016; Deo and Deshmukh, 2019). Recent microbiome studies have examined the diversity of bacterial communities using pyrosequencing of the 16S rRNA gene and have compared their functional profiles (Esberg et al., 2022; Narita and Kodama, 2022). Therefore, such sequencing can comprehensively investigate the characteristics of the oral microbiome and help us to understand the nature of periodontitis in elderly individuals.

The oral microbiome composition differs among various microhabitats (Caselli et al., 2020; Li et al., 2022). The prevalence of the most abundant bacterial genera differs in microbial distribution among sites [saliva, oral rinse (saliva after rinsing), tongue, and supra- and subgingival plaque] (Caselli et al., 2020; Li et al., 2022). As saliva samples are in contact with most surfaces (oral cavity) (Marsh et al., 2016; Saito et al., 2021), they may be suitable for evaluating the entire oral condition. As dental plaque induces oral inflammation, plaque samples are advantageous for assessment of site specificity. The tongue is the largest microbial habitat in the oral cavity, and various kinds of microbial species, both aerobic and anaerobic, inhabit the tongue coating. Tongue samples can be assessed using tongue coatings. The oral rinse microbiome is similar to the saliva sample but it is more representative of the whole site-specific microbiome than saliva samples (Caselli et al., 2020). Caselli et al. showed that several highly prevalent bacterial genera (*Streptococcus*, *Candidatus*, *Cutibacterium*, *Gemella*, *Pseudomonas*, *Actinomyces*, *Pseudopropionibacterium*, *Aggregatibacter*, *Corynebacterium*, *Staphylococcus*, *Veillonella*, *Parvimonas*, and *Micrococcus*) were more abundantly detected in oral rinse than in saliva (Caselli et al., 2020). Therefore, a representative oral microbiome may be suitable for assessment using oral rinse samples.

Previous studies have investigated the characteristics of the microbiome based on the presence or progression of periodontitis. However, there is insufficient information regarding the oral microbiome associated with the severity of periodontal conditions. First, the features of the oral microbiome in elderly Japanese individuals with ≥20 related with severity of periodontal condition are unclear. In particular, the features of the oral microbiome in elderly people who can achieve the goals of the 8020 Campaign (keeping 20 teeth or more at 80 years of age) associated with the severity of periodontal disease are not known. Second, only a few studies have assessed the oral microbiome by considering the number of teeth. Takeshita et al. showed that the number of teeth is related to the saliva microbiome (Takeshita et al., 2016). The number of teeth should be considered in the saliva and oral rinse microbiome surveys. Third, the method of assessment of the periodontal condition changed in July 2018 (Papapanou et al., 2018; Tonetti et al., 2018). The severity and complexity of periodontitis are divided into four stages (stage I/II is non-severe, and stage III/IV is severe periodontitis). Other large-scale studies of periodontal microbiota in the elderly used the 1999 classification system, which assessed only probing depth (PD) and clinical attachment level (CAL) (Papapanou et al., 2018). Therefore, we focused on the characteristics of the microbiome associated with non-severe periodontal conditions in subjects with ≥20 teeth. We hypothesized that certain characteristics of the oral microbiome could result in the maintenance of a substantial number of remaining teeth with a non-severe periodontal condition. The purpose of the present study was to compare the oral microbiome profile and diversity between elderly Japanese individuals (≥20 teeth) with a non-severe periodontal condition (stage I or II periodontitis) and a severe periodontal condition (stage III or IV periodontitis).

## 2 Methods

### 2.1 Study population

This cross-sectional study included patients who were receiving periodontal maintenance treatment every three months at the Clinic of Preventive Dentistry at Okayama University Hospital between December 2020 and January 2021. The participants had periodontitis and received periodontal maintenance treatment every three months for a minimum of 2 years (mean ± standard deviation, 10.2 ± 4.3 years). All participants underwent oral examination and oral rinse sampling, completed self-reported questionnaires, and had their body mass index (BMI) and body fat percentage determined. The inclusion criteria were age ≥60 years and ≥20 teeth. The exclusion criteria were the use of antibiotics within six months and regular use of mouthwash.

### 2.2 Ethical procedures and informed consent

The study was approved by the ethics committees of the Okayama University Graduate School of Medicine, Dentistry, and Pharmaceutical Sciences and Okayama University Hospital (no. 2104-017). All participants provided verbal and written informed consent to participate in this study.

### 2.3 Oral examination

Periodontitis was classified into four stages using the World Workshop on the Classification of Periodontal and Peri-Implant Diseases and Conditions (Papapanou et al., 2018; Tonetti et al., 2018). Well-experienced dentists investigated the probing depth, mobility, clinical attachment levels, and furcation involvement (Lindhe and Nyman, 1975; Nyman et al., 1975). Two dentists (NT and YN) checked the degree of bone loss on the X-rays. Medical records were used to check for tooth extraction for periodontitis. One dentist (NT) determined the stage of periodontitis (**Supplemental Table S1**). Following the classification of periodontitis, stages I and II were defined as the non-severe group, and stages III and IV were defined as the severe group (**Supplemental Table S1**). Stages I and II do not have tooth loss caused by periodontitis, interdental clinical attachment level at the site of greatest loss >4 mm, radiographic bone loss >33% root length, or maximum probing depth >5 mm (Papapanou et al., 2018; Tonetti et al., 2018).

### 2.4 Questionnaires

Self-reported questionnaires were used to investigate age, sex, smoking and alcohol status, number of snacks, use of interdental brushes and dental floss, mental stress, drugs, medical history, last instance of tooth brushing, and final intake of food, which are factors that can affect the composition of the oral microbiome (Cornejo Ulloa et al., 2019). Participants were also asked if they had used antibiotics within 6 months and if they regularly used mouthwash.

### 2.5 Oral rinse microbiome analysis

Oral rinse samples were collected to investigate the oral microbiome composition. To reduce the effects of food intake and tooth brushing, samples were collected at 10:00 AM–12:00 PM or 2:00 PM–4:00 PM, 1 h or more after food intake and tooth brushing. The participants rinsed with 3 ml of sterilized water for 10 s and then spat into a sterilized tube (Jo et al., 2019) to prevent contamination. After sampling, the samples were stored at –80°C.

DNA sequencing was performed according to the 16S metagenomics sequencing library preparation protocol (16s-metagenomic-library-prep-guide-15044223-b.pdf, 2021) of the Oral Microbiome Center (Taniguchi Dental Clinic, Kagawa, Japan). First, the V3 and V4 regions of the 16S rDNA were amplified using the primers 341F (5’-TCGTCGGCAGCGTCAGATGTGTATAAGAGACAGNNNCCTACGGGNGGCWGCAG-3’) and 806R (5’-GTCTCGTGGGCTCGGAGATGTGTATAAGAGACAGNNNGACTACHVGGGTATCTAATCC-3’). Next, adapter and index arrays were added to both sides of the DNA. The concentrations of the products were adjusted to be the same. Next-generation sequencing (NGS) was performed using the obtained sequences and the MiSeq platform (MiSeq Reagent V3 600 cycles, Illumina, San Diego, CA, USA). The read length was 300 bp × 2 paired ends. The obtained reads showed a cluster density of 923 K/mm^2^, a passing filter of 89.53%, and an average Q30 of 70.5%. After sequencing, the reads were refined using UPARSE (Edgar, 2013). The quality of the reads was checked using the FastQC software (The Babraham Institute, Babraham, Cambridgeshire, UK). Then, we removed low-quality portions of the lead ends (trimming) and combined the high-quality reads using Usearch. Then, NNN, which was inserted into the first PCR primer, was trimmed for improved cluster identification. Low-quality reads were removed using a qc filter (e-value >2.0; read length >200 bp). The refined reads were then clustered at 97% identity using UCLUST (Edgar, 2010), and chimera sequences were eliminated (Haas et al., 2011). Finally, the arrangements were collated using the Human Oral Microbiome Database (HOMD) (Chen et al., 2010), and bacterial species were identified.

### 2.6 Other factors

As BMI and body fat percentage are related to periodontitis, BMI and body fat percentage were measured using a body composition meter with a stature meter (BH-300A-N, TANITA, Tokyo, Japan).

### 2.7 Statistical analysis

To analyze the oral rinse microbiome, alpha (richness and evenness of bacterial taxa within a community) and beta (the ecological distance between samples) diversities were analyzed. Alpha diversities were assessed using Shannon and Simpson indices. Beta diversities were assessed using principal coordinate analysis (PCoA) based on weighted and unweighted UniFrac distances. Adonis and Anosim were used to assess differences in composition between the non-severe and severe groups. Permdisp2 was used to assess the differences in dispersion.

Network analysis was performed to investigate correlations among bacterial species at the species level. Correlations were evaluated using Pearson’s correlation coefficients, and correlation coefficients ≤|0.55| were shown (Sisk-Hackworth et al., 2021). Taxa are represented as nodes, with taxa abundance as node size, and edges as positive and negative associations. Blue nodes indicate the bacterial species related to the non-severe group, and red nodes are those related to the severe group. Positive correlations are represented by blue lines and negative correlations are indicated by yellow lines. The diversity indices and network analysis were analyzed using Calypso (http://cgenome.net:8080/calypso-8.84/faces/uploadFiles.xhtml; accessed on July 15, 2021).

To identify bacterial species in the non-severe group, the linear discriminant analysis effect size (LDA score) was calculated, with a cut-off value of 3.0. Galaxy (https://huttenhower.sph.harvard.edu/galaxy/; accessed on July 15, 2021) was used to calculate the LDA score.

Fisher’s exact test was used to examine differences in the presence of bacterial species between the non-severe and severe groups.

Statistical analyses were conducted using SPSS version 22 (IBM, Tokyo, Japan) for Fisher’s exact test and the Mann–Whitney *U* test. Statistical significance was set at P <0.05. To adjust for multiple comparisons, the q-value was calculated using the Benjamini and Hochberg false discovery rate based on the results of the p-value; all q-values <0.05 were considered significant.

## 3 Results

### 3.1 Participants’ characteristics

**Figure 1:**
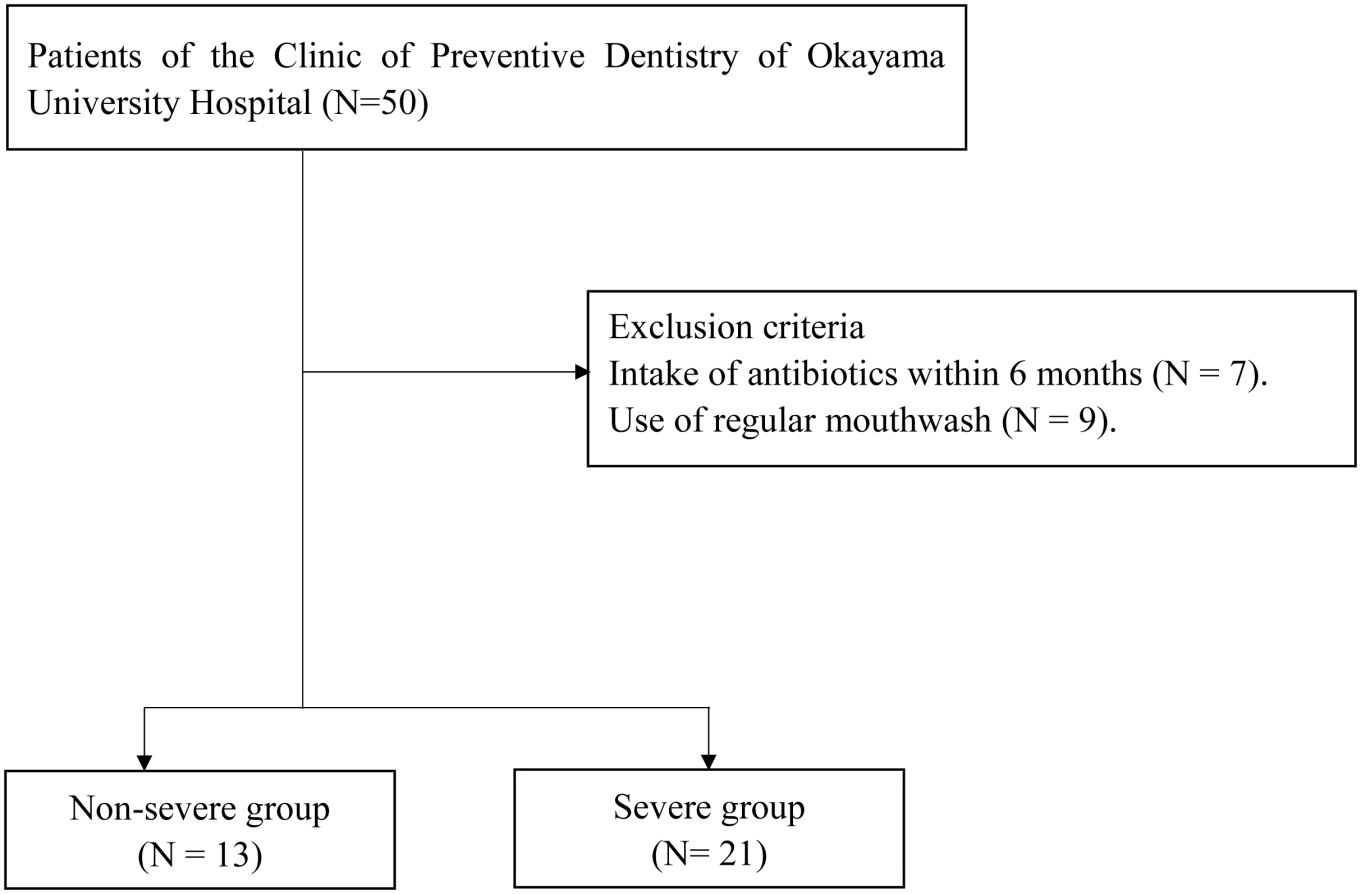
Flow chart.

**Figure 1** shows the flowchart of the present study. Fifty patients were included in this study. As 16 patients met the exclusion criteria, 34 patients (13 periodontally non-severe and 21 severe) were analyzed. No parameters were significantly different between the two groups (**Table 1**). The durations of periodontal maintenance treatment were not different between the two groups (non-severe group, 10.6 ± 4.4 years; severe group, 10.0 ± 4.3 years; p = 0.649 on the Mann–Whitney *U* test, data not shown).

**Table 1:** Differences in parameters between the non-severe and severe groups.

		Non-severe group	Severe group	P
		N = 13	N = 21	
Sex	Male	5 (38.5)^a^	8 (38.1)	1^b^
	Female	8 (61.5)	13 (61.9)	
Diabetes mellitus	No	11 (84.6)	19 (90.5)	0.627
	Yes	2 (15.4)	2 (9.5)	
Smoking status	No	5 (38.5)	7 (33.3)	1
	Past	8 (61.5)	14 (66.7)	
Alcohol	No	6 (46.2)	12 (57.1)	0.756
	Past	1 (7.7)	2 (9.5)	
	Current	6 (46.2)	7 (33.3)	
Snack	No	6 (46.2)	5 (23.8)	0.288
	1 time/day	4 (30.8)	12 (57.1)	
	≥2 times/day	3 (23.1)	4 (19.0)	
Interdental brushes	No	2 (15.4)	1 (4.8)	0.518
	Sometimes	3 (23.1)	7 (33.3)	
	Everyday	8 (61.5)	13 (61.9)	
Dental floss	No	9 (69.2)	13 (61.9)	0.910
	Sometimes	3 (23.1)	6 (28.6)	
	Everyday	1 (7.7)	2 (9.5)	
Mental stress	No	4 (30.8)	11 (52.4)	0.086
	Sometimes	9 (69.2)	7 (33.3)	
	Always	0 (0)	3 (14.3)	
Age, y		71 (66, 74)^c^	73 (67, 79)	0.400^d^
Remaining teeth		27 (26, 28)	26 (24, 27)	0.112
Body mass index, kg/m^2^		23.3 (18.4, 25.0)	22.3 (20.7, 23.6)	0.897
Body fat percentage		25.2 (14.4, 37.1)	28.9 (21.8, 32.8)	0.982
Time from last tooth brushing (min)		145 (60, 225)	120 (60, 180)	0.576
Time from last food intake (min)		180 (135, 225)	180 (125, 205)	0.889

^a^N (%), ^b^Fisher’s exact test, ^c^Median (25% confidence interval, 75% confidence interval); ^d^Mann–Whitney U test.

### 3.2 Oral rinse microbiota

#### 3.2.1 Diversity

**Figure 2:**
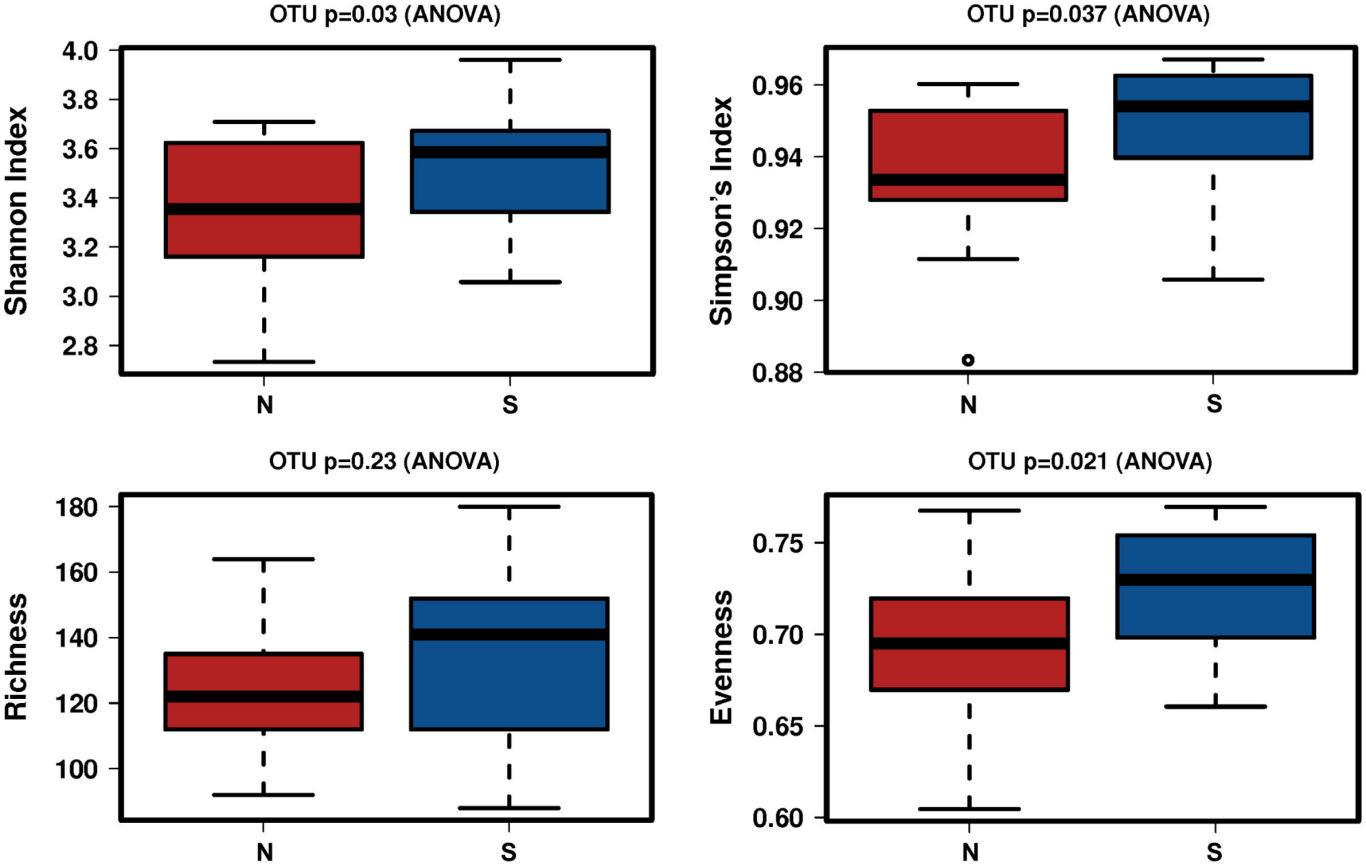
The difference in the alpha diversity index between the non-severe group and the severe group. N, non-severe group; S, severe group; OTU, operational taxonomic unit.

**Figure 3:**
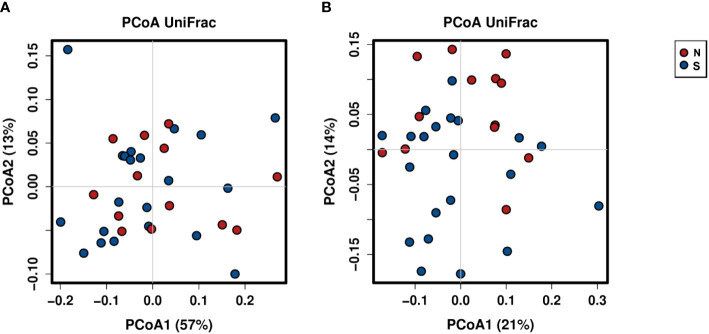
Principal coordinate analysis of the non-severe group and the severe group based on weighted **(A)** and unweighted **(B)** UniFrac distances. The plots are based on weighted **(A)** and unweighted **(B)** UniFrac distances and the relative abundances of bacterial OTUs. Red and blue plots indicate the non-severe and severe groups, respectively. N, non-severe group; S, severe group; PCoA, principal coordinate analysis.

A total of 2,941,777 reads were merged, and 2,806,083 reads were filtered from 34 oral rinse samples. A total of 463 operational taxonomic units (OTUs) were obtained based on 97% sequence similarity, and 11 phyla, 23 classes, 34 orders, 54 families, 100 genera, and 353 species were identified. In alpha diversity, the Shannon and Simpson’s indices were significantly greater in the periodontitis group than those in the non-severe group (**Figure 2**). Evenness, but not richness, was significantly lower in the non-severe group than that in the severe group (**Figure 2**). All parameters of beta diversity were not significantly different between the non-severe and severe groups (weighted UniFrac distance (Anosim, P = 0.77; Adonis, P = 0.459; Permdisp2, P = 0.71); unweighted UniFrac distance (Anosim, P = 0.151; Adonis, P = 0.0583; Permdisp2, P = 0.588)) (**Figure 3**).

#### 3.2.2 Difference in relative abundance

**Figure 4:**
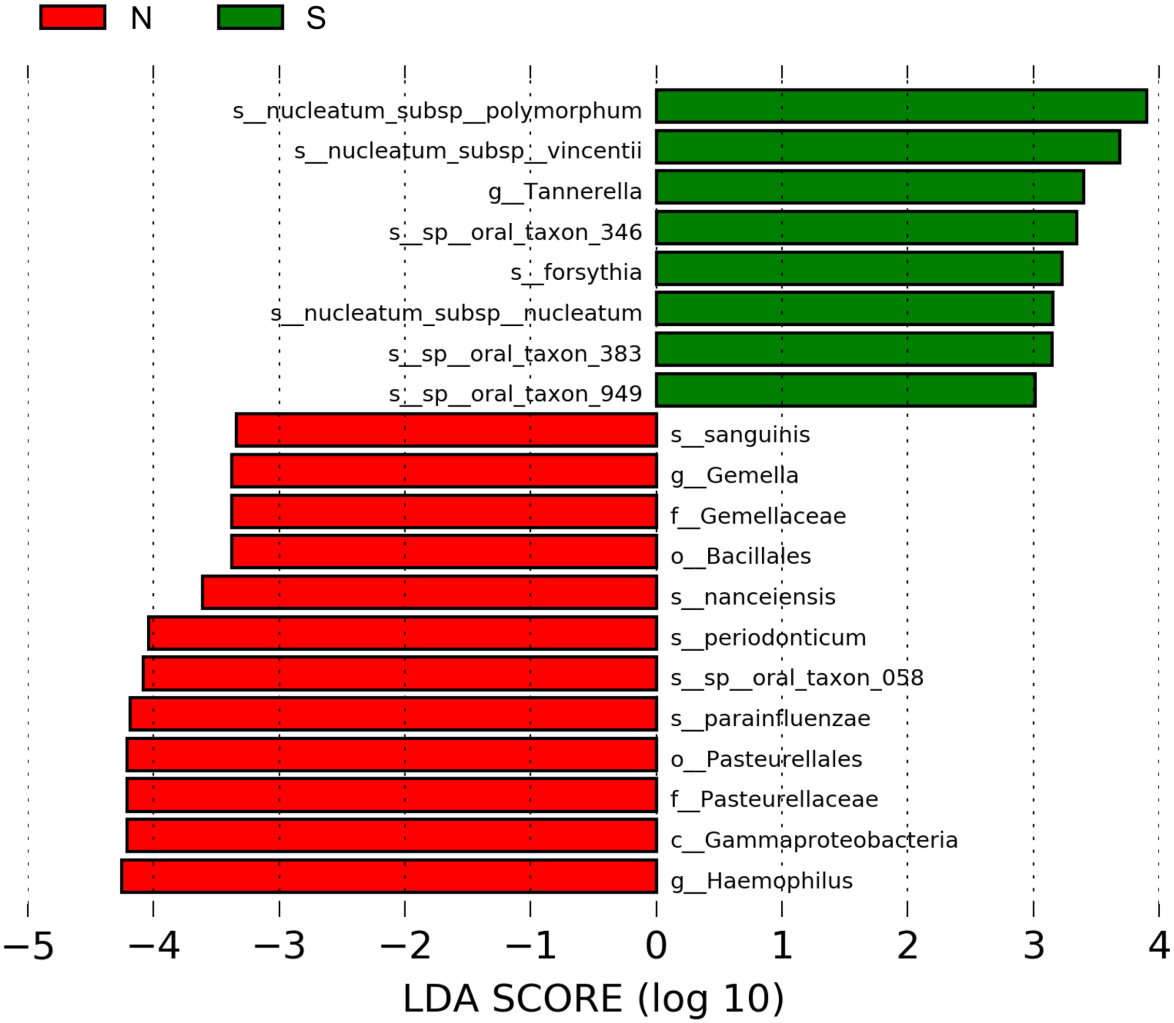
Graphics of linear discriminant analysis (LDA) effect size (LEfSe) for non-severe and severe groups. The horizontal bars indicate the effect size of each taxon. The length of the bar shows the log10 transformed LDA score, as indicated by the vertical dotted lines. The green and red bars indicate the non-severe and severe groups, respectively. The threshold of the logarithmic LDA score for the discriminative features was set to 3.0. The taxon of bacteria with a significant change (p <0.05) in relative abundance is shown along the horizontal lines. The taxon level is abbreviated as p, phylum; c, class; o, order; f, family; g, genus; and s, species; N, non-severe group; S, severe group.

**Figure 4** shows the results of the LDA effect size (comparison of relative abundances). One class, two orders, two families, two genera, and five species in the non-severe group had LDA scores >3.0. Four of the five species were significantly more common in the non-severe group than in the severe group (*Prevotella nanceiensis* (*P. nanceiensis*), *Gemella sanguinis* (*G. sanguinis*), *Fusobacterium periodonticum* (*F. periodonticum*), and *Haemophilus parainfluenzae* (*H. parainfluenzae*)) (**Table 2**).

**Table 2:** Comparison of the relative abundance of bacterial species between the non-severe and severe groups.

	Non-severe group (n = 13)	Severe group (n = 21)	p^a^	q^b^
*Prevotella nanceiensis*	0.0064 (0.0029, 0.0221)^c^	0.0018 (0.0002, 0.0039)	0.005	0.025
*Gemella sanguinis*	0.0074 (0.0036, 0.0096)	0.0034 (0.0022, 0.0054)	0.014	0.035
*Streptococcus* sp. *oral taxon 058*	0.0837 (0.0656, 0.1000)	0.0581 (0.0500, 0.0756)	0.050	0.050
*Fusobacterium periodonticum*	0.0549 (0.0262, 0.0810)	0.0215 (0.0131, 0.0519)	0.035	0.044
*Haemophilus parainfluenzae*	0.0534 (0.0294, 0.0898)	0.0246 (0.0139, 0.0544)	0.029	0.048

^a^Mann–Whitney U test, ^b^adjusted p-value by Benjamini–Hochberg’s false discovery rate (FDR), ^c^Median (25th percentile, 75th percentile).

#### 3.2.3 Difference in existence

**Supplemental Figure S1** shows the bacterial species with a prevalence of >70% in the non-severe or severe group. Seven bacterial species (*Actinomyces* sp. oral taxon 170, *Lachnospiraceae [G-2]* sp. oral taxon 096, *Stomatobaculum* sp. oral taxon 097, *Leptotrichia* sp. oral taxon 215, *Leptotrichia* sp. oral taxon 221, *Haemophilus sputorum*, and *TM7 [G-1]* sp. oral taxon 347) with a prevalence of >70% were observed only in the non-severe group. However, the prevalence of the seven bacterial species was not significantly different between the two groups (**Supplemental Table S2**). These results indicate that the presence of bacterial species did not differ between the two groups.

#### 3.2.4 Network analysis

**Figure 5:**
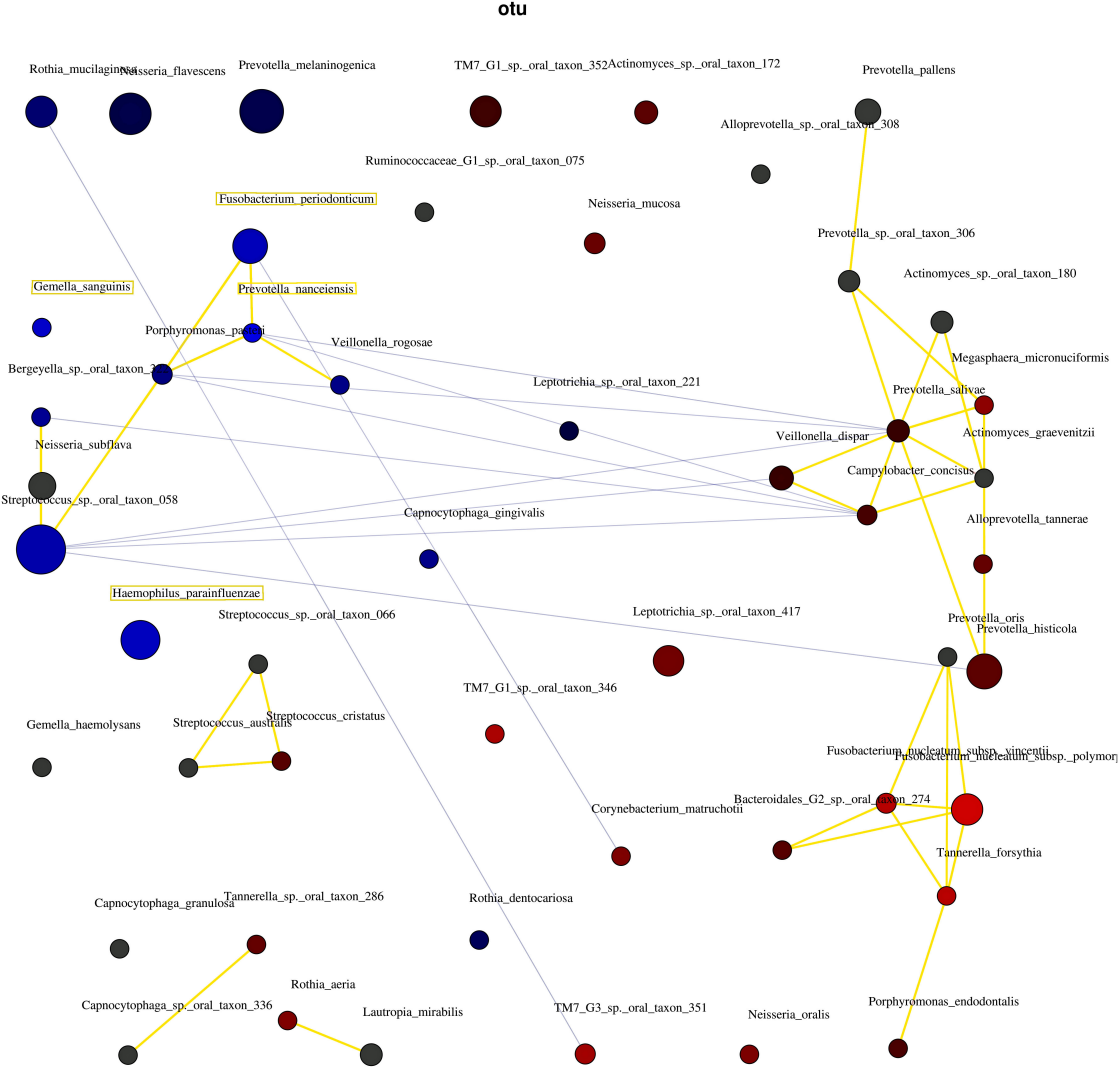
The interaction of bacteria using network analysis. Taxa and explanatory variables are represented as nodes, taxa abundance as node size, and edges as positive and negative associations. The networks were generated using Spearman’s rho. The nodes of positively or negatively correlated taxa are connected with yellow and blue edges, respectively. The blue and red nodes indicate the bacteria related to the non-severe and severe groups, respectively. Positive and negative correlations as edges are indicated by yellow and blue lines, respectively.

**Figure 5** shows the correlations between the bacterial species. The three network groups were as follows: Group 1, *P. nanceiensis*, *F. periodonticum*, *Neisseria subflava*, *Streptococcus* sp. oral taxon 058, *Bergeyella* sp. oral taxon 322, *Porphyromonas pasteri*, and *Veillonella rogosae*; Group 2, *Prevotella oris*, *Fusobacterium nucleatum* subsp. vincentii, *Fusobacterium nucleatum* subsp. polymorphum, *Bacteroidales [G-2]* sp. oral taxon 274, *Tannerella forsythia*, and *Porphyromonas endodontalis*; and Group 3, *Prevotella pallens*, *Prevotella* sp. oral taxon 306, *Actionomyces* sp. oral taxon 180, *Megasphaera micronuciformis*, *Prevotella salivae*, *Veillonella disper*, *Actinomyces graevenitzii*, *Alloprevotella tannerae*, and *Prevotella histicola*. A negative correlation was observed between Groups 1 and 2.

## 4 Discussion

To the best of our knowledge, this is the first study to investigate the characteristics of the oral microbiome in elderly Japanese people with ≥20 teeth with a non-severe periodontal condition and compare them to those with severe periodontitis. The present study focused on the diversity and specific species of bacteria in non-severe periodontal conditions. Evenness differed significantly between the non-severe and severe groups. The relative abundances of four bacterial species (*P. nanceiensis*, *G. sanguinis*, *F. periodonticum*, and *H. parainfluenzae*) were found to be greater in the non-severe group than those in the severe group. Seven bacterial species with a prevalence of >70% were found only in the non-severe group. However, the prevalence of the seven bacterial species was not significantly different between the two groups.

In the present study, the alpha diversity was significantly lower in the non-severe group than in the severe group. Some studies have shown higher alpha diversity in patients with severe periodontal disease than in non-severe and healthy controls (Griffen et al., 2012; López-Martínez et al., 2020). Saito et al. showed that alpha diversity was correlated with periodontitis severity in the saliva and plaque (Saito et al., 2021). However, Plachokova et al. showed that the alpha diversity of plaque was not associated with the severity of periodontitis (Plachokova et al., 2021). Previous studies did not incorporate the association between alpha diversity and periodontal status because the techniques used, standardization methods, and sample sizes varied (Deo and Deshmukh, 2019). Although the findings remain controversial, the major trends of previous studies support the present study showing that alpha diversity was low in the non-severe group and suggested that elderly people with ≥20 teeth and a non-severe periodontal condition had lower alpha diversity.

Microbiome structure (beta diversity) did not differ between the non-severe and severe groups. This result suggests that the microbial community composition in the oral rinse did not differ in the severity of periodontitis. Saito et al. (2021) showed no difference in beta diversity by severity of periodontitis. The present observation supports that of previous studies and indicates that microbial community composition might not differ between non-severe and severe groups in elderly Japanese people.

*P. nanceiensis* was isolated in 2007 (Alauzet et al., 2007) and is related to a healthy airway (Hilty et al., 2010); and is a representative bacterium of healthy airways (Hilty et al., 2010). However, it is unclear whether *P. nanceiensis* affects periodontal conditions. *P. nanceiensis* has saccharolytic effects and produces acid as a major metabolic end-product (Alauzet et al., 2007). *P. gingivalis* does not increase under acidic conditions (Takahashi and Schachtele, 1990). In the present study, *P. nanceiensis* was negatively correlated with *P. gingivalis* (Spearman’s correlation coefficient, −0.403; p = 0.018; data not shown). It is possible that *P. nanceiensis* inhibits the growth of *P. gingivalis*.

*G. sanguinis* was more common in the non-severe group than in the severe group.*G. sanguinis* produces alkaline phosphatases (Ulger-Toprak et al., 2010). Alkaline phosphatase hydrolyzes non-organic pyrophosphate, which inhibits the mineralization process (Coleman, 1992). Alkaline phosphatase activity is upregulated in regenerating human periodontal cells (Kuru et al., 1999). *G. sanguinis* tends to inhibit the growth of *P. gingivalis* (Miyoshi et al., 2021), because it decreases the pH due to acid production, which inhibits the growth of *P. gingivalis* (Miyoshi et al., 2021). Thus, the genus *Gemella* may also inhibit periodontitis.

*F. periodonticum* was more common in the non-severe group than in the severe group. *F. periodonticum* belongs to the so-called “orange complex,” which is thought to include the precursor pathogens in the establishment of a “red complex” ecology, which leads to periodontal destruction (Socransky and Haffajee, 2005). A previous study showed that *F. periodonticum* was present in subjects with chronic periodontitis (Ximenez-Fyvie et al., 2006). Recently, *F. periodonticum* has been considered as a potential commensal with opportunistic disease potential (Park et al., 2010). *Fusobacterium* is a middle colonizer found in the gingival sulcus and periodontal pockets (Periasamy and Kolenbrander, 2010). The non-severe group included stages I and II, so it is possible that the relative abundance of *F. periodonticum*, which belongs to the “orange complex,” was greater than that in the severe group, which included stages III and IV. Therefore, *F. periodonticum* may not be associated with the severity of periodontal conditions.

*H. parainfluenzae* in a non-severe periodontal condition are more abundant than in a severe condition (Relvas et al., 2021). Van Hoogmoed et al. (2008) showed that *H. parainfluenzae* reduces the adhesion of *P. gingivalis*. *P. gingivalis*, which is a late colonizer, adheres to other bacterial species to upregulate periodontitis (Periasamy and Kolenbrander, 2009), but *H. parainfluenzae* can inhibit this adhesion. Thus, *H. parainfluenzae* may contribute to non-severe periodontal conditions.

These four bacterial species may interact to regulate the severity of periodontal conditions. Abusleme et al. (2021) and Relvas et al. (2021) showed a network analysis of health-associated communities. Both studies showed that bacteria related to the severity of periodontitis interact. In the present study, network analysis confirmed the interaction between the bacterial species in the non-severe group (**Figure 5**). The results indicated that the four bacterial species co-occurred and led to the non-severe periodontal condition.

The results of this study are therefore clinically relevant. First, the present results are useful for estimating ideal periodontal status. The participants in the present study had ≥20 teeth (average number of teeth: 25.9) and were aged ≥60 years. Therefore, the present results show the pattern of a non-severe microbiome in elderly people. Secondly, an oral rinse microbiome was observed in the two groups. Since oral rinse samples are easy to collect, clinicians may be able to easily determine whether their patients are at risk of periodontitis and tooth loss.

The strength of the present study was that confounders were controlled. Several factors affect the oral microbiome (Cornejo Ulloa et al., 2019). It was possible to control for some of the factors that affect the oral microbiome, including age, sex, diet, smoking, drugs, stress, access to dental care, and oral hygiene habits (**Table 1**). The present study may have reduced the effects of confounders that affected the composition of the oral microbiome.

Some limitations of this study must be considered when interpreting the results. First, it was not possible to adjust for nutritional factors, salivary cortisol, buffer capacity, salivary pH, attachment surfaces, or body temperature (Cornejo Ulloa et al., 2019). All participants were ≥60 years old, and most people in this age category had a chronic disease. However, the effects of medical history (other than diabetes mellitus) were not evaluated. These factors may affect the salivary microbiome. Second, since the present study was a cross-sectional study, it is unclear whether health-related bacterial species can improve or prevent periodontitis. Third, evaluation of the oral microbiome may be underestimated in periodontitis. As all participants received treatments every three months, the oral health conditions of the participants might be better than those of the general population. However, since the saliva microbiome recovers one month after periodontal treatment (Greenwood et al., 2020), oral rinse sample collection at 3 months following periodontal treatment might not have affected the present results. Fourth, since the sample size was small, it is possible that the power of statistical testing was low (type 2 error might be large). A future study with a larger sample size might show that more bacterial species are related to non-severe periodontal conditions.

In conclusion, the oral microbiome of elderly Japanese individuals showed lower alpha diversity in the non-severe group than in the severe group. Additionally, *P. nanceiensis*, *G. sanguinis*, *F. periodonticum*, and *H. parainfluenzae* were significantly more abundant in the non-severe group than those in the severe group. These bacterial species may interact to regulate non-severe periodontal conditions. However, the beta diversity did not differ between the two groups.

## Data availability statement

The datasets presented in this article are not readily available because the ethical application form, which is approved, is not describe about data registration to public database. Requests to access the datasets should be directed to Naoki Toyama, pu171qxi@s.okayama-u.ac.jp.

## Ethics statement

This study was reviewed and approved by the Ethics Committees of Okayama University Graduate School of Medicine, Dentistry and Pharmaceutical Sciences and Okayama University Hospital. The patients/participants provided their written informed consent to participate in this study.

## Author contributions

NT contributed to the conceptualization, methodology, formal analysis, investigation, resources, data curation, writing (original draft), and funding acquisition. DE contributed to the conceptualization, methodology, investigation, resource writing, review, and editing. AY, DF, NS, YN, MN, and IS contributed to the investigation, resources, and writing, review, and editing. MI contributed to the writing, review, and editing of the manuscript. MM contributed to the investigation, resources, writing-review and editing, supervision, and project administration. All authors contributed to the article and approved the submitted version.

## Funding

This research was funded by the FUTOKU Foundation from April 2020 to March 2021.

## Conflict of interest

The authors declare that the research was conducted in the absence of any commercial or financial relationships that could be construed as a potential conflict of interest.

## Publisher’s note

All claims expressed in this article are solely those of the authors and do not necessarily represent those of their affiliated organizations, or those of the publisher, the editors and the reviewers. Any product that may be evaluated in this article, or claim that may be made by its manufacturer, is not guaranteed or endorsed by the publisher.

## Supplementary material

Supplementary Figure 1 The bacteria with a prevalence >70% in the non-severe group or the severe group. Non-severe group only: Bacteria observed in >70% of patients in the non-severe group and in <70% of patients in the severe group. Non-severe > Severe: Bacteria observed in both groups with a prevalence >70%, and the prevalence was greater in the non-severe group than that in the severe group. Non-severe = Severe: Bacteria observed in both groups with a prevalence >70% and were the same. Severe > Non-severe: Bacteria observed in both groups with a prevalence >70%, and the prevalence was lower in the non-severe group than that in the severe group. Severe group only: Bacteria observed in >70% of patients in the severe group and <70% of patients in the non-severe group.

## References

[B1] 16s-metagenomic-library-prep-guide-15044223-b.pdf (2021). 16S metagenomic sequencing library preparation. Available at: https://support.illumina.com/documents/documentation/chemistry_documentation/16s/16s-metagenomic-library-prep-guide-15044223-b.pdf (Accessed September 3, 2021).

[B2] 8020 Promotion Foundation (2021). Available at: https://www.8020zaidan.or.jp (Accessed September 3, 2021).

[B3] Abusleme L. Hoare A. Hong B.-Y. Diaz P. I. (2021). Microbial signatures of health, gingivitis, and periodontitis. Periodontology 86, 57–78. doi: 10.1111/prd.12362 33690899

[B4] Alauzet C. Mory F. Carlier J.-P. Marchandin H. Jumas-Bilak E. Lozniewski A. Y. (2007). Prevotella nanceiensis sp. nov., isolated from human clinical samples. Int. J. Syst. Evol. Microbiol. 57, 2216–2220. doi: 10.1099/ijs.0.65173-0 17911286

[B5] Caselli E. Fabbri C. D’Accolti M. Soffritti I. Bassi C. Mazzacane S.. (2020). Defining the oral microbiome by whole-genome sequencing and resistome analysis: the complexity of the healthy picture. BMC Microbiol. 20, 120. doi: 10.1186/s12866-020-01801-y 32423437 PMC7236360

[B6] Chen T. Yu W.-H. Izard J. Baranova O. V. Lakshmanan A. Dewhirst F. E. (2010). The human oral microbiome database: A web accessible resource for investigating oral microbe taxonomic and genomic information. Database (Oxford) 2010, baq013. doi: 10.1093/database/baq013 20624719 PMC2911848

[B7] Chen M. X. Zhong Y. J. Dong Q. Q. Wong H. M. Wen Y. F. (2021). Global, regional, and national burden of severe periodontiti 1990–2019: An analysis of the global burden of disease study 2019. J. Clin. Periodontol. 48, 1165–1188. doi: 10.1111/jcpe.13506 34101223

[B8] Coleman J. E. (1992). Structure and mechanism of alkaline phosphatase. Annu. Rev. Biophys. Biomol. Struct. 21, 441–483. doi: 10.1146/annurev.bb.21.060192.002301 1525473

[B9] Cornejo Ulloa P. van der Veen M. H. Krom B. P. (2019). Review: modulation of the oral microbiome by the host to promote ecological balance. Odontology 107, 437–448. doi: 10.1007/s10266-019-00413-x 30719639 PMC6732124

[B10] Deo P. N. Deshmukh R. (2019). Oral microbiome: Unveiling the fundamentals. J. Oral. Maxillofac. Pathol. 23, 122–128. doi: 10.4103/jomfp.JOMFP_304_18 PMC6503789 31110428

[B11] Dewhirst F. E. Chen T. Izard J. Paster B. J. Tanner A. C. R. Yu W.-H.. (2010). The human oral microbiome. J. Bacteriol. 192, 5002–5017. doi: 10.1128/JB.00542-10 20656903 PMC2944498

[B12] Edgar R. C. (2010). Search and clustering orders of magnitude faster than BLAST. Bioinformatics 26, 2460–2461. doi: 10.1093/bioinformatics/btq461 20709691

[B13] Edgar R. C. (2013). UPARSE: Highly accurate OTU sequences from microbial amplicon reads. Nat. Methods 10, 996–998. doi: 10.1038/nmeth.2604 23955772

[B14] Esberg A. Eriksson L. Johansson I. (2022). Site- and time-dependent compositional shifts in oral microbiota communities. Front. Oral. Health 3. doi: 10.3389/froh.2022.826996 PMC8921071 35300180

[B15] Greenwood D. Afacan B. Emingil G. Bostanci N. Belibasakis G. N. (2020). Salivary microbiome shifts in response to periodontal treatment outcome. Proteomics Clin. Appl. 14, 2000011. doi: 10.1002/prca.202000011 32223062

[B16] Griffen A. L. Beall C. J. Campbell J. H. Firestone N. D. Kumar P. S. Yang Z. K.. (2012). Distinct and complex bacterial profiles in human periodontitis and health revealed by 16S pyrosequencing. ISME J. 6, 1176–1185. doi: 10.1038/ismej.2011.191 22170420 PMC3358035

[B17] Haas B. J. Gevers D. Earl A. M. Feldgarden M. Ward D. V. Giannoukos G.. (2011). Chimeric 16S rRNA sequence formation and detection in Sanger and 454-pyrosequenced PCR amplicons. Genome Res. 21, 494–504. doi: 10.1101/gr.112730.110 21212162 PMC3044863

[B18] Hilty M. Burke C. Pedro H. Cardenas P. Bush A. Bossley C.. (2010). Disordered microbial communities in asthmatic airways. PloS One 5, e8578. doi: 10.1371/journal.pone.0008578 20052417 PMC2798952

[B19] Hoogmoed C. G. V. Geertsema-doornbusch G. I. Teughels W. Quirynen M. Busscher H. J. der Mei H. C. V. (2008). Reduction of periodontal pathogens adhesion by antagonistic strains. Oral. Microbiol. Immunol. 23, 43–48. doi: 10.1111/j.1399-302X.2007.00388.x 18173797

[B20] Jo R. Nishimoto Y. Umezawa K. Yama K. Aita Y. Ichiba Y.. (2019). Comparison of oral microbiome profiles in stimulated and unstimulated saliva, tongue, and mouth-rinsed water. Sci. Rep. 9, 16124. doi: 10.1038/s41598-019-52445-6 31695050 PMC6834574

[B21] Kuru L. Griffiths G. S. Petrie A. Olsen I. (1999). Alkaline phosphatase activity is up regulated in regenerating human periodontal cells. J. Periodontal Res. 34, 123–127. doi: 10.1111/j.1600-0765.1999.tb02231.x 10207841

[B22] Li X. Liu Y. Yang X. Li C. Song Z. (2022). The oral microbiota: Community composition, influencing factors, pathogenesis, and interventions. Front. Microbiol. 13. doi: 10.3389/fmicb.2022.895537 PMC9100676 35572634

[B23] Lindhe J. Nyman S. (1975). The effect of plaque control and surgical pocket elimination on the establishment and maintenance of periodontal health. A longitudinal study of periodontal therapy in cases of advanced disease. J. Clin. Periodontol. 2, 67–79. doi: 10.1111/j.1600-051X.1975.tb01727.x 1055729

[B24] López-Martínez J. Chueca N. Padial-Molina M. Fernandez-Caballero J. A. García F. O’Valle F.. (2020). Bacteria associated with periodontal disease are also increased in health. Med. Oral. Patol. Oral. Cir. Bucal 25, e745–e751. doi: 10.4317/medoral.23766 32701927 PMC7648922

[B25] Mark Welch J. L. Rossetti B. J. Rieken C. W. Dewhirst F. E. Borisy G. G. (2016). Biogeography of a human oral microbiome at the micron scale. Proc. Natl. Acad. Sci. U.S.A. 113, E791–E800. doi: 10.1073/pnas.1522149113 26811460 PMC4760785

[B26] Marsh P. D. Do T. Beighton D. Devine D. A. (2016). Influence of saliva on the oral microbiota. Periodontology 70, 80–92. doi: 10.1111/prd.12098 26662484

[B27] Miyoshi T. Oge S. Nakata S. Ueno Y. Ukita H. Kousaka R.. (2021). Gemella haemolysans inhibits the growth of the periodontal pathogen porphyromonas gingivalis. Sci. Rep. 11, 11742. doi: 10.1038/s41598-021-91267-3 34083694 PMC8175725

[B28] Narita Y. Kodama H. (2022). Identification of the specific microbial community compositions in saliva associated with periodontitis during pregnancy. Clin. Oral. Invest. 26, 4995–5005. doi: 10.1007/s00784-022-04468-z 35352183

[B29] Nazir M. A. (2017). Prevalence of periodontal disease, its association with systemic diseases and prevention. Int. J. Health Sci. (Qassim) 11, 72–80. 28539867 PMC5426403

[B30] Nielsen S. J. Trak-Fellermeier M. A. Joshipura K. Dye B. A. (2016). Dietary fiber intake is inversely associated with periodontal disease among US Adults12. J. Nutr. 146, 2530–2536. doi: 10.3945/jn.116.237065 27798338 PMC5118764

[B31] Nyman S. Rosling B. Lindhe J. (1975). Effect of professional tooth cleaning on healing after periodontal surgery. J. Clin. Periodontol. 2, 80–86. doi: 10.1111/j.1600-051X.1975.tb01728.x 1094035

[B32] Palinkas M. Borges T. de F. Junior M. T. Monteiro S. A. C. Bottacin F. S.. (2019). Alterations in masticatory cycle efficiency and bite force in individuals with periodontitis. Int. J. Health Sci. (Qassim) 13, 25–29. PMC6392481 30842715

[B33] Papapanou P. N. Sanz M. Buduneli N. Dietrich T. Feres M. Fine D. H.. (2018). Periodontitis: Consensus report of workgroup 2 of the 2017 world workshop on the classification of periodontal and peri-implant diseases and conditions. J. Periodontol. 89, S173–S182. doi: 10.1002/JPER.17-0721 29926951

[B34] Park S.-N. Park J.-Y. Kook J.-K. (2010). Development of speciels-specific polymerase chain reaction primers for detection of fusobacterium periodonticum. Microbiol. Immunol. 54, 750–753. doi: 10.1111/j.1348-0421.2010.00279.x 21223363

[B35] Periasamy S. Kolenbrander P. E. (2009). Mutualistic biofilm communities develop with porphyromonas gingivalis and initial, early, and late colonizers of enamel. J. Bacteriol. 191, 6804–6811. doi: 10.1128/JB.01006-09 19749049 PMC2772475

[B36] Periasamy S. Kolenbrander P. E. (2010). Central role of the early colonizer veillonella sp. in establishing multispecies biofilm communities with initial, middle, and late colonizers of enamel. J. Bacteriol. 192, 2965–2972. doi: 10.1128/JB.01631-09 20154130 PMC2901697

[B37] Peterson J. Garges S. Giovanni M. McInnes P. Wang L. Schloss J. A.. (2009). The NIH human microbiome project. Genome Res. 19, 2317–2323. doi: 10.1101/gr.096651.109 19819907 PMC2792171

[B38] Plachokova A. S. Andreu-Sánchez S. Noz M. P. Fu J. Riksen N. P. (2021). Oral microbiome in relation to periodontitis severity and systemic inflammation. Int. J. Mol. Sci. 22, 5876. doi: 10.3390/ijms22115876 34070915 PMC8199296

[B39] Relvas M. Regueira-Iglesias A. Balsa-Castro C. Salazar F. Pacheco J. J. Cabral C.. (2021). Relationship between dental and periodontal health status and the salivary microbiome: Bacterial diversity, co-occurrence networks and predictive models. Sci. Rep. 11, 929. doi: 10.1038/s41598-020-79875-x 33441710 PMC7806737

[B40] Saito S. Aoki Y. Tamahara T. Goto M. Matsui H. Kawashima J.. (2021). Oral microbiome analysis in prospective genome cohort studies of the tohoku medical megabank project. Front. Cell Infect. Microbiol. 10. doi: 10.3389/fcimb.2020.604596 PMC7878372 33585276

[B41] Sisk-Hackworth L. Ortiz-Velez A. Reed M. B. Kelley S. T. (2021). Compositional data analysis of periodontal disease microbial communities. Front. Microbiol. 12, 617949. doi: 10.3389/fmicb.2021.617949 34079525 PMC8165185

[B42] Socransky S. S. Haffajee A. D. (2005). Periodontal microbial ecology. Periodontology 38, 135–187. doi: 10.1111/j.1600-0757.2005.00107.x 15853940

[B43] Survey of Dental Diseases (2017). Available at: https://www.mhlw.go.jp/toukei/list/62-17.html (Accessed September 6, 2021).

[B44] Takahashi N. Schachtele C. F. (1990). Effect of pH on the growth and proteolytic activity of porphyromonas gingivalis and bacteroides intermedius. J. Dent. Res. 69, 1266–1269. doi: 10.1177/00220345900690060801 2191980

[B45] Takeshita T. Kageyama S. Furuta M. Tsuboi H. Takeuchi K. Shibata Y.. (2016). Bacterial diversity in saliva and oral health-related conditions: the hisayama study. Sci. Rep. 6, 22164. doi: 10.1038/srep22164 26907866 PMC4764907

[B46] Tanji F. Komiyama T. Ohi T. Hattori Y. Watanabe M. Lu Y.. (2020). The association between number of remaining teeth and maintenance of successful aging in Japanese older people: A 9-year longitudinal study. Tohoku J. Exp. Med. 252, 245–252. doi: 10.1620/tjem.252.245 33162454

[B47] Tonetti M. S. Greenwell H. Kornman K. S. (2018). Staging and grading of periodontitis: Framework and proposal of a new classification and case definition. J. Periodontol. 89, S159–S172. doi: 10.1002/JPER.18-0006 29926952

[B48] Ulger-Toprak N. Summanen P. H. Liu C. Rowlinson M.-C. Finegold S. M. Y. (2010). Gemella asaccharolytica sp. nov., isolated from human clinical specimens. Int. J. Syst. Evol. Microbiol. 60, 1023–1026. doi: 10.1099/ijs.0.001966-0 19666813

[B49] Ximenez-Fyvie L. A. Almaguer-Flores A. Jacobo-Soto V. Lara-Cordoba M. Sanchez-Vargas L. O. Alcantara-Maruri E. (2006). Description of the subgingival microbiota of periodontally untreated Mexican subjects: Chronic periodontitis and periodontal health. J. Periodontol. 77, 460–471. doi: 10.1902/jop.2006.050177 16512761

[B50] Yoshihara A. Watanabe R. Nishimuta M. Hanada N. Miyazaki H. (2005). The relationship between dietary intake and the number of teeth in elderly Japanese subjects. Gerodontology 22, 211–218. doi: 10.1111/j.1741-2358.2005.00083.x 16329229

