# Supplementary figures and images for: Features of the oral microbiome in Japanese elderly people with 20 or more teeth and a non-severe periodontal condition during periodontal maintenance treatment: A cross-sectional study
Source: Front Cell Infect Microbiol. 2022 Oct 6;12:957890. doi: 10.3389/fcimb.2022.957890 (PMC9582337; doi:10.3389/fcimb.2022.957890)

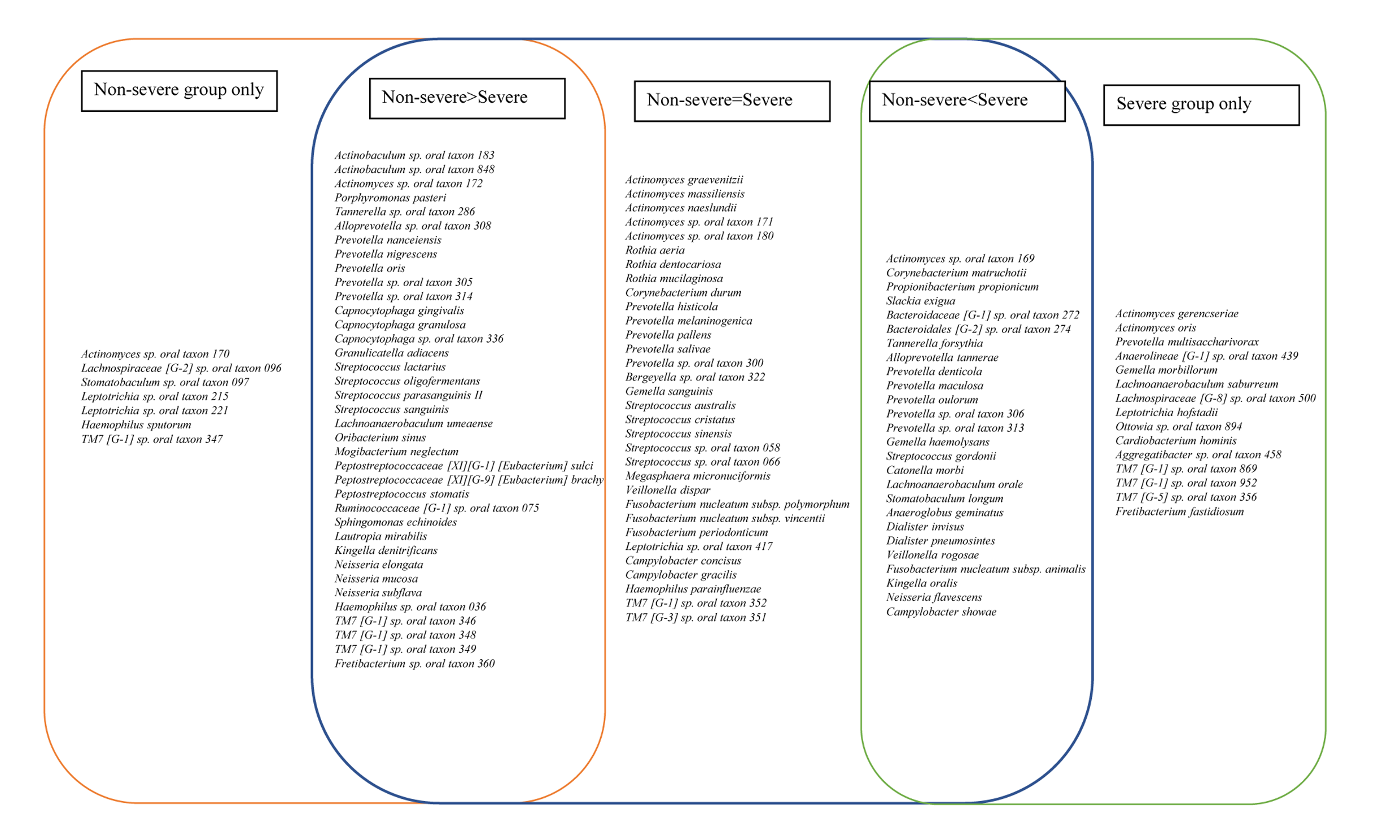

Supplement: Supplementary Figure 1 — The bacteria with a prevalence >70% in the non-severe group or the severe group. Non-severe group only: Bacteria observed in >70% of patients in the non-severe group and in <70% of patients in the severe group. Non-severe > Severe: Bacteria observed in both groups with a prevalence >70%, and the prevalence was greater in the non-severe group than that in the severe group. Non-severe = Severe: Bacteria observed in both groups with a prevalence >70% and were the same. Severe > Non-severe: Bacteria observed in both groups with a prevalence >70%, and the prevalence was lower in the non-severe group than that in the severe group. Severe group only: Bacteria observed in >70% of patients in the severe group and <70% of patients in the non-severe group. [file Image_1.tif]
