# Supplementary material for: Features of the oral microbiome in Japanese elderly people with 20 or more teeth and a non-severe periodontal condition during periodontal maintenance treatment: A cross-sectional study
Source: Front Cell Infect Microbiol. 2022 Oct 6;12:957890. doi: 10.3389/fcimb.2022.957890 (PMC9582337; doi:10.3389/fcimb.2022.957890)
Supplement: Supplementary file 2 [file Table_1.docx]

| Supplementary Table S1. Classification of periodontitis based on stages defined by severity | | | | | |
| --- | --- | --- | --- | --- | --- |
| **Group** | | **Non-severe group** | | **Severe group** | |
| **Periodontal stage** | | **Stage Ⅰ** | **Stage Ⅱ** | **Stage Ⅲ** | **Stage Ⅳ** |
| **Severity** | **Interdental CAL at site of greatest loss** | 1 to 2 mm | 3 to 4 mm | ≥5 mm | ≥5 mm |
|  | **Radiographic bone loss** | Coronal third  (<15%) | Coronal third  (15% to 33%) | Extending to mild-third of root and beyond | Extending to mild-third of root and beyond |
|  | **Tooth loss** | No tooth loss due to periodontitis | | Tooth loss due to periodontitis of ≤4 teeth | Tooth loss due to periodontitis of ≥5 teeth |
| **Complexity** | **Local** | Maximum probing depth ≤4 mm  Mostly horizonal bone loss | Maximum probing depth ≤5 mm  Mostly horizonal bone loss | In addition to stage Ⅱ complexity:  Probing depth ≥6 mm  Vertical bone loss ≥3 mm  Furcation involvement Class Ⅱ or Ⅲ  Moderate ridge defect | In addition to stage Ⅲ complexity:  Need for complex rehabilitation due to:  Masticatory dysfunction  Secondary occlusal trauma (tooth mobility degree ≥2)  Severe ridge defect  Bite collapse, drifting, flaring  Less than 20 remaining teeth (10 opposing pairs) |

Tonetti, M. S., Greenwell, H., and Kornman, K. S. (2018). Staging and grading of periodontitis: Framework and proposal of a new classification and case definition. *Journal of Periodontology* 89, S159–S172. doi: 10.1002/JPER.18-0006.
