# Supplementary material for: Features of the oral microbiome in Japanese elderly people with 20 or more teeth and a non-severe periodontal condition during periodontal maintenance treatment: A cross-sectional study
Source: Front Cell Infect Microbiol. 2022 Oct 6;12:957890. doi: 10.3389/fcimb.2022.957890 (PMC9582337; doi:10.3389/fcimb.2022.957890)
Supplement: Supplementary file 3 [file Table_2.docx]

| Supplementary Table S2. Comparison of the bacteria present between the non-severe and severe groups | | | |  |
| --- | --- | --- | --- | --- |
|  | Non-severe group  (n = 13) | Severe group  (n = 21) | p ^a^ | q ^b^ |
| *Actinomyces sp. oral taxon 170* | 12 (92.3) ^c^ | 12 (57.1) | 0.029 | 0.174 |
| *Lachnospiraceae [G-2] sp. oral taxon 096* | 11 (84.6) | 13 (61.9) | 0.158 | 0.474 |
| *Stomatobaculum sp. oral taxon 097* | 10 (76.9) | 13 (61.9) | 0.363 | 0.436 |
| *Leptotrichia sp. oral taxon 215* | 10 (76.9) | 12 (57.1) | 0.241 | 0.362 |
| *Leptotrichia sp. oral taxon 221* | 11 (84.6) | 13 (61.9) | 0.158 | 0.474 |
| *Haemophilus sputorum* | 10 (76.9) | 13 (61.9) | 0.363 | 0.437 |
| *TM7 [G-1] sp. oral taxon 347* | 10 (76.9) | 14 (66.7) | 0.524 | 0.524 |
| ^a^ Fisher's exact test, ^b^ adjusted p value by Benjamini-Hochberg’s false discovery rate (FDR), ^c^ N (%) | | | |  |
